# Adoption knowledge: a citizen-scientific use of *FamilySearch* to understand Peruvian adoption

**DOI:** 10.1080/25729861.2022.2123635

**Published:** 2022-10-24

**Authors:** Jessaca Leinaweaver, Milagros Caroline Forrester

**Affiliations:** a Anthropology, Brown University, Providence RI, USA; b Independent Scholar, London, UK

**Keywords:** Adoption, citizen science, documents, genealogy, Peru, Adoção, ciência-cidadã, documentos, genealogia, Peru, Adopción, ciencia ciudadana, documentos, genealogía, Perú

## Abstract

This article analyzes the citizen-scientific work conducted by a Peruvian adoptee, co-author Milagros Caroline Forrester, who is collecting, archiving, coding, and analyzing the birth records of Peruvian adoptees she encounters through her ongoing review of FamilySearch, a depository of genealogical data run by the Mormon Church. Forrester’s project resembles the personal birthparent searches conducted by many adopted people, but at another scale: becoming “citizen science” through an enlargement of the sample size to hundreds of cases, an analytic approach to the form of each document and what meta-communicative data the form provides, and a determination of the implications and significance of the synthesized results. In her research with birth registries, Forrester was inspired by scientific methodologies in the social sciences, including database creation, management, and analysis, and open coding of textual artifacts. This article shows that the nuance that laypeople bring to personally implicated citizen science can lead to engaged and meaningful findings that reach communities not often heard from in scholarly research. Forrester’s study is grounded in an awareness of her fellow adoptees’ challenges in finding information about their origins, and ambivalence about the significance of those findings.

## Conhecimento adotivo: Uma aproximação científico-cidadão de *FamilySearch* para a compreensão do sistema peruano de adoções

**RESUMO:**

Este artigo analisa o trabalho científico-cidadão realizado por uma peruana adotada, a coautora deste ensaio, Milagros Caroline Forrester, que está coletando, arquivando, codificando e analisando os registros de nascimento de pessoas adotadas peruanas que ela encontra através de sua revisão contínua do FamilySearch, um depósito de dados genealógicos administrados pela Igreja Mórmon. O projeto de Forrester se assemelha, em outra escala, às buscas que muitas pessoas adotadas empreendem pelos seus pais biológicos. Desta maneira, pode-se afirmar que devido à sua metodologia, uma ampliação do tamanho da amostra para centenas de casos, uma abordagem analítica do formato de cada documento e quais dados metacomunicativos o formulário fornece, e uma determinação das implicações e significado dos resultados sintetizados, a pesquisa de Forrester é uma forma de “ciência cidadã.” As metodologias científicas nas quais Forrester se inspirou para sua pesquisa com registros de nascimento provêm das ciências sociais, incluindo criação, gerenciamento e análise de banco de dados e codificação aberta de artefatos textuais. Este artigo mostra que a nuance que os pesquisadores leigos trazem para a uma ciência cidadã, dotada de uma perspectiva pessoal, pode levar a descobertas engajadas e significativas que atingem comunidades que muitas vezes não são consideradas em pesquisas acadêmicas. O estudo de Forrester baseia-se na consciência dos desafios que seus companheiros adotados enfrentam para encontrar informações sobre suas origens e na ambivalência sobre o significado dessas descobertas.

## Conocimiento adoptivo: el abordaje científico-ciudadano de *FamilySeach* para entender la adopción peruana

**RESUMEN:**

Este artículo analiza el trabajo científico-ciudadano realizado por la co-autora Milagros Caroline Forrester, una persona adoptada de origen peruana, durante su recopilación, archivo, codificación y análisis de los registros de nacimiento de personas peruanas adoptadas en *FamilySearch*, un depositorio de datos genealógicos gestionado por la iglesia mormona. El trabajo de Forrester es similar al realizado por muchas personas adoptadas que buscan sus datos de nacimiento, pero en una escala que la convierte en “ciencia ciudadana” por la ampliación de la muestra a cientos de casos, el análisis de la forma de cada documento y su meta-mensaje, así como por una determinación de las implicaciones y la significación de los resultados producidos. En su investigación de los registros de nacimiento, Forrester se inspiró en las metodologías científicas de las ciencias sociales incluyendo la creación, gestión y análisis de bases de datos y la codificación abierta de productos textuales. Este artículo muestra cómo la perspectiva de personas fuera de la academia pero implicadas personalmente en la ciencia ciudadana puede conducir a hallazgos significativos y comprometidos, capaces de llegar a comunidades a las que no suele llegar la investigación académica. El estudio de Forrester se basa en la concientización por la que atravesaron sus compañeros adoptados ante los desafíos de la búsqueda de sus orígenes y la ambivalencia del significado de esos hallazgos.

## 1. Introduction

Documents are so often precious to adopted people. They are one of the few material objects that connect the adopted person to their earliest moments of personal history (Kim 2019). However, laws in many countries prevent disclosure of adoptees’ original birth certificates, apparently motivated to protect the privacy of the birth mother, to exorcise potential stigmas relating to ideologies about legitimacy, and to strengthen the adoptive tie by excluding previous connections (Yngvesson and Mahoney 2000, 86, Leinaweaver 2019, 8). In the absence of an original birth certificate, other scraps of information take on heightened importance in adoptees’ searches (Yngvesson 2003; Mariner 2020, 10). Bringing together such documents that are significant on an individual level can yield answers to different kinds of questions about adoptees as an incipient community.

FamilySearch, a depository of genealogical data run by the Mormon Church, may be an under-utilized resource in this field. Genealogy is highly significant to Mormons, who believe their deceased kin may be saved through baptism by proxy, if they are properly traced through genealogical records. The Church’s vast bricks-and-mortar genealogical library, assembled in connection with this imperative (Cannell 2005, 345), also supports FamilySearch, a freely available online resource for ancestry research worldwide. Family-Search’s public facing language is largely sanitized of Mormon overtones, benignly advising that it “safeguards more than 3.5 billion images on microfilm, microfiche, and digital media. Our purpose is simple: to help people connect with their ancestors through easy access to historical records.”^1^

These records include birth certificates, which can bear muted traces of bureaucratic adoption processes (Kim 2019, 457; see also Pearson 2021). In Peru, a “birth certificate” (*Acta de Nacimiento*) is “a legal instrument that identifies a person by name, grants them Peruvian nationality, and recognizes their parentage, among other things.”^2^ Thus, “birth certificates” are also used to record other related demographic or civic events that fit unevenly into a biography, like claiming paternity, registering a birth long after the fact (see Leinaweaver 2008, 150 on ‘claiming maternity’), or adoption.^3^ In current Peruvian adoption law, once an adoption has been approved by administrative resolution, that approval is transmitted to RENIEC (Peru’s civil registry) “causing the original birth certificate to be voided and a new birth certificate to be recorded and produced, not using the term ‘adopted child.’”^4^ What we explore here is not the traces of individual adoptions, full information about which is still in most cases inaccessible, but rather how through personally-motivated citizen science, an inchoate adoptive community is recoverable from the presence of adoptive birth certificates in FamilySearch.

This article analyzes the citizen-scientific work conducted by a Peruvian adoptee, co-author Milagros Caroline Forrester, who is collecting, archiving, coding, and analyzing the birth records of Peruvian adoptees she encounters through her ongoing review of FamilySearch. It is a project she can see herself in: a home-grown, personally meaningful activity (see Invernizzi 2020, 230) resonant with birthparent-searches (see, e.g. Yngvesson 2003; Yngvesson and Mahoney 2000; Carsten 2000; Kim 2019) that becomes “citizen science” through an enlargement of the sample size to hundreds of cases, an analytic approach to the form of each document and what meta-communicative data the form provides (compare Gordillo 2006; Ellison 2018), and a determination of the implications and significance of the synthesized results.

Forrester’s work exemplifies how citizen science can be a project of *democratization* (Irwin 1995; see also Invernizzi 2020, 246): because of personal interest and commitment, she is better able to resist the repeated assertion in the work of trained demographers (e.g. Kane 1993; Selman 2002) that data from states of origin are simply unreachable. Invernizzi categorizes certain forms of public participation in science as emergent “at the margins and interstices … They result from the absence, in mainstream research agendas, of issues relevant to social groups … and also, as part of alternative social projects and ideologies which demand knowledge and technologies that are compatible with them” (2020, 242). In her and her peers’ own absence from the statistics she pored over, Forrester located what Frickel and colleagues call “undone science” - an area of potential research with a social benefit that is nonetheless ignored or uncompleted by mainstream science (2010, 445). She therefore is questioning the assumptions behind choices that inform demographic studies, identifying data sources and analyzing them, both individually, to identify important elements that appear upon qualitative study, and collectively, to look for patterns and changes over time and across regions. In addition, through outreach to credentialed scholars like Leinaweaver and others,^5^ she is attempting to promote more expansive research agendas.

This project then resonates with the assessment of science and public participation outlined by Bucchi and Neresini, who assert that “Lay knowledge is not an impoverished or quantitatively inferior version of expert knowledge; it is qualitatively different. Factual information is only one ingredient of lay knowledge, in which it interweaves with other elements (value judgments, trust in the scientific institutions, the person’s perception of his or her ability to put scientific knowledge to practical use) to form a corpus no less sophisticated than specialist expertise” (2008, 451). As part of this interweaving, Forrester has, on her own, come to some of the research practices that family demographers use - drawing on a massive, publicly available dataset in order to discover patterns of demographic behavior.^6^ In that sense, her project also shows the appeal, to citizens, of the *idea* of “big data” to answer persistent questions many adoptees have about where they came from, and how many others are like them. As befits the sheer quantity of birth registries available in the system, the scientific methodologies Forrester was inspired by for her research were centered in the social sciences, including database creation, management, and analysis, and open coding of textual artifacts. This article first describes her project and locates it in the literature, before considering two areas of her analysis – the fine-grained consideration of the documents themselves, and the synthetic work Forrester did to identify trends and themes.

## 2. Context: origins of the project

Forrester, who was adopted from Peru at sixteen months of age, recalls that “My adoptive mum had all of our [my and my siblings’] files. They were green file folders and she had all of them in chronological order … It was an archive of our lives … We had to look at it when she was in front of us – we were not allowed to take it away. It became very precious to us. At the age of 17 my file, with all my adoption papers and documents, was handed to me – basically I was responsible to preserve them and understand my documentation.” These early lessons in the importance of caring for and honoring identity documents took root (compare Carsten 2000, 691; Kim 2019, 451), and shaped Forrester’s later encounters with FamilySearch.

Forrester recalls that she first came across FamilySearch in 2013 “whilst trying to search for information related to my birth mom.” She had previously conducted a birthparent search in the “traditional” or analog way, traveling to Peru so she could make in-person requests for adoption files, orphanage documents, and hospital papers, but unfortunately her birth mother had died before they could meet. Later, sitting in her bedroom, searching Google for information and typing in her birth mother’s full name to FamilySearch, she found a birth certificate, and was amazed both at how easy it was to understand, and at how comprehensive the available information was. The certificate offered personal information, including Forrester’s birth grandparents’ names, her birth mother’s place of birth – an hacienda – and her birth mother’s race, “indígena” or Indigenous. It soon became clear to Forrester that FamilySearch, which included freely available Peruvian birth registry data, had interesting potential for adoptees, who often face multiple obstacles in learning more about their birth family histories. These moments of discovery are not unlike the moving experience of Mormon genealogists who feel a spiritual joy at tracking down hard-to-locate relatives (Cannell 2005, 346). Yet there were barriers here too – Forrester tried to search for her own original birth certificate, but discovered that Mormon genealogists had not acquired the birth records from all Peruvian departments (a unit similar to states), and of those records that were present, not all of them were indexed (i.e. made easily searchable by a volunteer having transcribed some of the key identifiers on the handwritten certificate).^7^ Amid a sea of documents, as she began to collect examples that sparked her interest, she thus also marked an ongoing absence – those documents that were redacted or are otherwise unavailable, including certificates for herself and others in her family.

**Figure 1.**
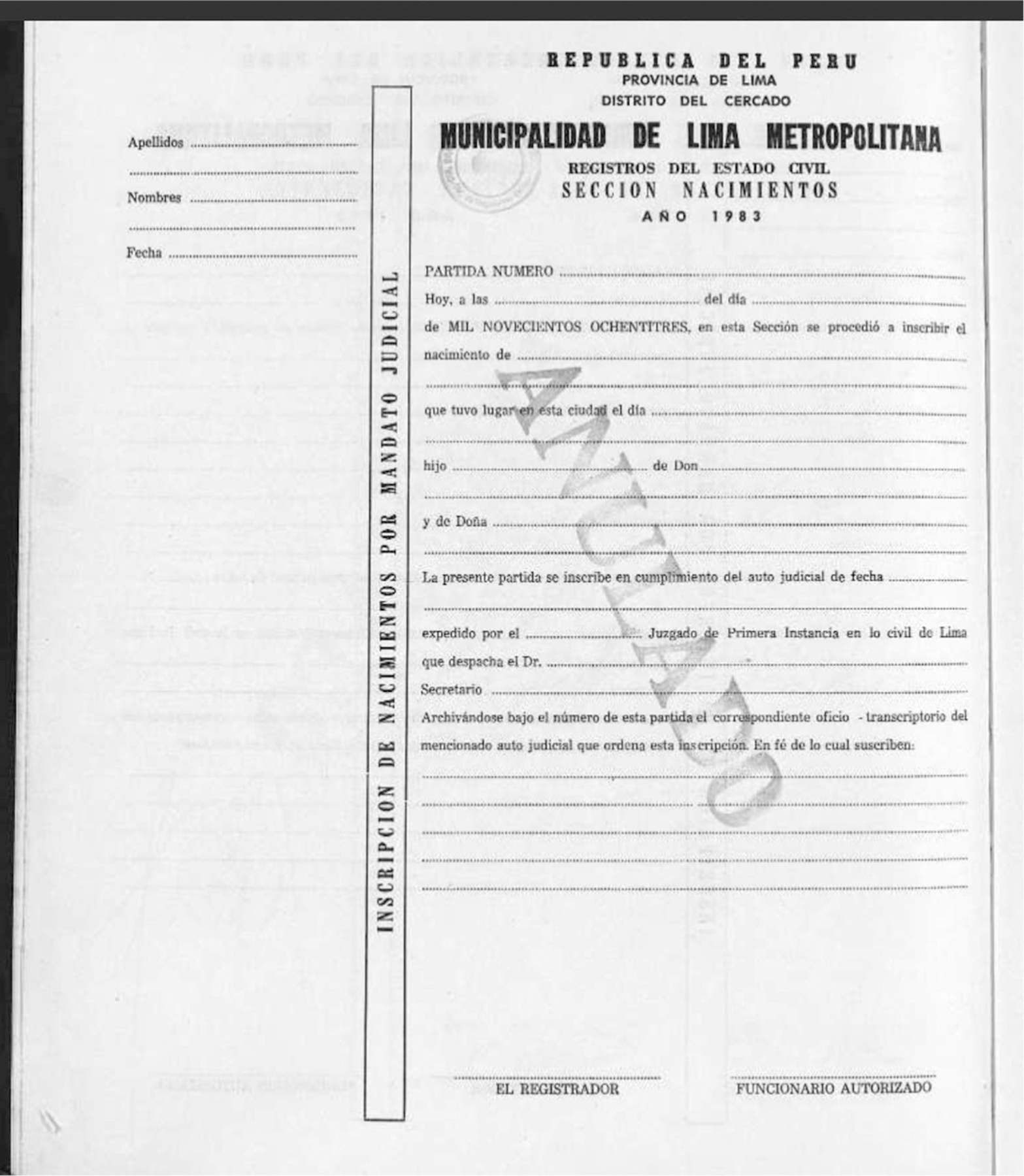
Blank, voided birth registration form. Municipality of Metropolitan Lima, 1983. Source: Collection of Forrester, originally downloaded from FamilySearch.org (http://www.familysearch.org: accessed 30 September 2021).

**Figure 2.**
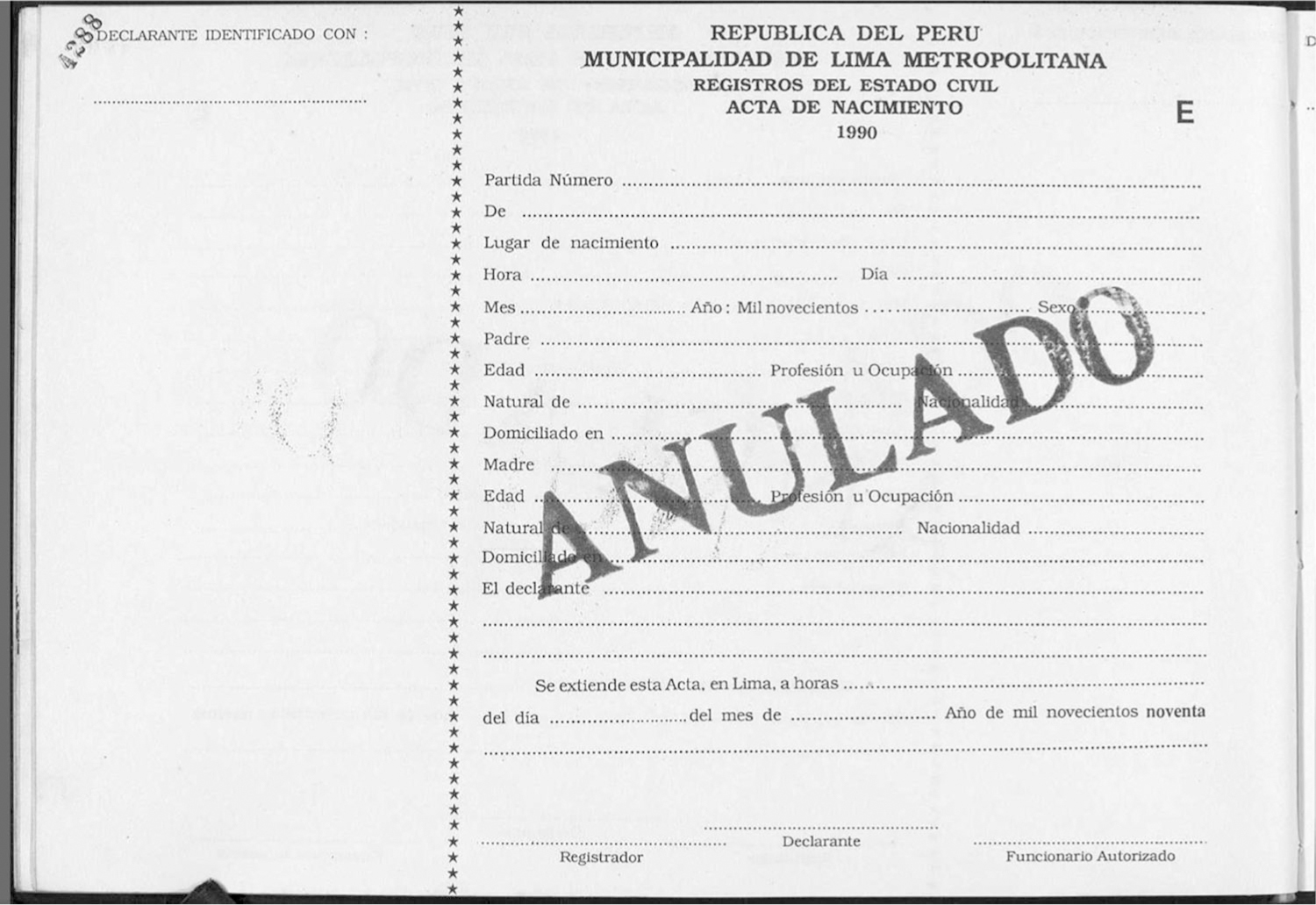
Blank, voided birth registration form. Municipality of Metropolitan Lima, 1990. Source: Collection of Forrester, originally downloaded from FamilySearch.org (http://www.familysearch.org, accessed 30 September 2021).

A couple years later, Forrester was trying to help a friend find their birth certificate.^8^ The documents from the friend’s birthplace were not indexed, so instead of being able to simply type in the name and have it pop up in FamilySearch, Forrester realized that in order to find it she would need to click through every scanned entry from the book of birth records for that year and town. In Peru during the period she became familiar with, birth records were entered, by hand, in books; different districts or regions often had different styles of recording, and collected different information, but the end result was a chronologically organized book containing all the registries that had been recorded in that particular location. As she clicked through the entries in the relevant book, seeking certificates that seemed to belong to adoptees, she observed that certificates had different features in different time periods and districts (see Figures 1 and 2). Over the next year or two, she casually reviewed random books on FamilySearch in no particular order whenever she had time, amassing what she estimates as 250 examples of certificates that denoted adoptees.

As Forrester writes in her reflections on this period, “Jump to 2020 and Covid-19 – my work schedule changed dramatically and I realised that I would actually have the time to develop this idea! I looked at the certificates that I had already and decided to formulate a consistent approach.” During the enforced months at home, and inspired by the data visualizations illustrating the daily BBC coronavirus news briefings, she wondered whether she could collect data and create charts, graphs, and analyses just like she saw unfold on screen each day. She organized her information in an Excel spreadsheet. She began by reviewing books from the 1990s from Lima as an entry point to try to inductively learn how FamilySearch was organized, how long it would take for her to review the contents of the book, and what information she would ultimately include in her spreadsheet.^9^ She developed a practice of completing the group of volumes that corresponded to a full year of registrations, and then proceeding to review the books in the next or the previous year. If a book yielded no entries, she still recorded it on her spreadsheet, and when she did find a relevant entry, she placed a hyperlink in her spreadsheet so she could directly jump back to the FamilySearch record. She then expanded to explore other regions and other decades, each one taking a little time to get adjusted to due to a different organization of the document or handwriting that was tough to decipher. Her general approach has been toward collecting, and comprehensive coverage – not necessarily representativeness but rather increasing the number of records within the limited amount of time she can devote to her “hobby.” The result is that her sampling strategy is purposive, not comprehensive, focusing on large, more populous cities where she knew that adoptions had taken place. As her intuitive recognition of the patterns in birth certificates and registration books deepened through her review, she ended up creating a home-made database that has many hundreds of entries. The unstructured free time that lockdown made possible enabled Forrester to embark on what she experienced as a personally meaningful quest to create and assemble historical data and knowledge where there had been none before – revealing through documentary, archival work the space that Peruvian adoptees had previously occupied in shadow.

## 3. Background and literature: (de)humanizing paper

While some forms of “citizen science” are reactive, involving laypeople responding to scientists’ calls for aid, others are proactive: everyday people adopting methodologies associated with the sciences in order to resolve experienced political problems (e.g. Polleri 2019). In this case, work done by scientists feels incomplete, as it does not encompass the personal experience of adoptees like Forrester, yet the models that their work follows are recognizably productive. Callon offers the example of patients suffering from “orphan diseases” that are so rare that they are frequently understudied by medical professionals. “To assert themselves and have their existence recognized, they naturally engage in what could be called a primitive accumulation of scientific knowledge … which puts both disease and victims into the same field of objective knowledge,” he explains, noting how such patients research diseases, collect biological and narrative data, and carry out observations (Callon 1999, 90). Similarly, Forrester collects and analyzes data that she finds to be inadequately accounted for by researchers of adoption.

The project is also situated in recent bio-digital understandings of community, ancestry, and heritage, which we trace in part to much earlier research on kinship, a traditional topic within cultural anthropology (Leinaweaver’s field of specialization). Kinship studies here both benefits from and nourishes approaches associated with STS that focus on the cultural valences of elevating particular forms of knowledge as scientific. As Van Wichelen notes, “Since the 1970s – though primarily in Europe and the US – the child’s right to know their biological origins has become increasingly accepted and valued” (2019, 348; see also Yngvesson and Mahoney 2000, 91; Blyth et al. 2009). Within this context, Forrester’s search for adoptees in this depository of genealogical knowledge consisting of thousands of digitized birth certificates is an example of “technologies of belonging,” which M’charek and colleagues frame as “the power of (other-directed) identification … [through] technological devices and bureaucratic procedures such as databases, lists, maps, border controls, and genetic tests” (2014, 463). This is comparable to the usages of DNA technologies to identify and claim ancestry, recognition, and rights (see, e.g. Nelson 2008; TallBear 2013; Abel and Tsosie 2019). For example, a genetic history project in Cape Town linked DNA to national history, marketing “genetic genealogical information as a way for participants to negotiate belonging through understanding the diverse genetic histories of the South African people … ‘where we come from and who we are’” (Foster 2016, 1016).^10^ Such tools and technologies, as they become more widely available, are being repurposed to midwife experiences of and understandings of biologized identity.

This project also, of course, highlights the very specific bureaucratic and technological processes that produced those adoptees, and their tangible outcomes: paperwork. As Flores notes, “neither documents nor processes can be fully understood without the other, since together they form the material and context through which people experience the parameters of institutional and civic inclusion” (2016, 543; see also Pellegrino 2022). The analysis of these documentary and processual elements of adoption contributes to literature on kinship more broadly, which itself becomes a novel contribution to STS. As Kim writes, “The overwriting of DNA’s ‘truths’ and the immediacy of consanguineous social relations by adoption paperwork … highlights paperwork’s ability to authorize certain relations and to disallow others through acts of legibility and illegibility. Paperwork …, like DNA, … contains information that mediates and also *produces* relatives” (2019, 473; compare Chelcea 2016; Ryan 2020; Mariner 2020). Adopted persons’ vital data may be recorded in civil registries; as Bledsoe observes, such material is “both tightly circumscribed by the state and highly deceptive … As people have attempted to use the register to forge new identities or hold old ones in reserve, bureaucrats have followed anxiously behind, trying to alter declarations of identity and, when necessary, to tread lightly on inconvenient political truths” (2010, 109). “Western” forms of adoption, with the walls they build around past identities through declarations about privacy, mean that knowledge about one’s own personal history must be *discovered* – which makes such knowledge constitutive, or identity-shifting (Carsten 2007, 408).

## 4. Undone demography of adoption

Forrester’s exploration of, production of, and engagement with data was partly sparked by an encounter with an edited book by demographer Peter Selman, *Intercountry Adoption: Development, trends, and perspectives* (2000). The implied breadth of its 30 chapters excited Forrester until she pored through it and found only one reference to Peru. This was part of the impetus that drove her to *find* Peruvian adoptees in other places. It was also an instance of a broader problem: a notable dearth of reliable data on adoptions, and substantial limitations on existing data (United Nations 2009: iii). Early attempts by demographers to calculate global numbers of international adoptions were regularly qualified by the observation that most sending countries did not keep data on adoptions (though see Kane 1993, 325 on exceptions, Colombia and Korea) and some receiving countries, including the UK, did not centralize their statistics (Kane 1993).^11^ Kane also observes that data from receiving countries is likely incomplete, too, because of reported practices of irregular adoptions, such as “‘births’ in sending countries by travelling prospective adoptive parents” (1993, 334; compare to more recent surrogacy practices for contracting parents from countries where surrogacy is illegal). Selman, noting “the difficulties involved in obtaining comparative data from many states of origin,” explains that he follows Kane (1993) and others in “using data gathered by receiving states to provide an estimate of the relative levels of intercountry adoption in states of origin” (2002, 213). Of course, states of origin have poor data in part because international adoption often is a consequence of war, disaster, or political upheaval, each of which also prevents or hampers efforts to collect data (Selman 2002; Briggs and Marre 2009).

However, the Internet has made possible new forms of data storage and access, a “digital shift” (Dickel and Franzen 2016, 10) which has happened alongside legal and social changes that tend to favor open access to personal biographical data (Wegar 1997). Carsten points to how rapidly being able to access a much broader range of kinship information and knowledge affects “the way we ‘do kinship,’ or our sense of self” (2007, 404). The form of FamilySearch’s database suggests two ways this operates: through a network orientation, encouraging users to scale up and consider themselves and their individual ancestry searches as part of a larger, more community-oriented project, and through framing crowdsourcing as a democratic act, contributing to a broader common good.

First, FamilySearch’s *network* orientation has analogues to DNA databases like 23andMe, personal genomics which imply “an understanding of self as always already a subject in the network” (Levina 2010, 1). FamilySearch’s materials, too, urge users to see themselves as connected to other users, with statements like “Save what you find about ancestors to their profile in Family Tree. What you save will be preserved and automatically shared with relatives who may be looking for the same information.”^12^ Augmenting others’ efforts to record genealogy, like uploading one’s DNA profile, is like “an act of citizenship, precisely because it is good for the network” (Levina 2010, 2). Secondly, there is also a *democratization* sensibility similar to “crowd science,” an attitude of being “able to count on an almost unlimited number of virtual staff” (Dickel and Franzen 2016, 4). FamilySearch exhorts users to “**Get Involved …** help make a difference for researchers around the world” through indexing: “a volunteer project that makes images of historical records – like census returns or obituaries – searchable online. It’s a great way to give back to the genealogy community and make family discoveries possible”^13^ (see also Cannell 2005, 346). Indexing is a form of “crowdsourcing,” or the use of digital infrastructure to delegate a task to “an anonymous crowd, who voluntarily perform the task” (Dickel and Franzen 2016, 3).^14^

Building on and honoring these principles of network orientation and democratization, Forrester shaped a collection, attempting to curate the broader archive of FamilySearch to give form to an inchoate imagined community of adoptees from Peru. Collecting these documents was not an easy task because adoptee birth certificates are nowhere pulled together all in one place, nor, in most cases, do they explicitly say that the individual being registered was adopted. In addition, there are notable differences in documentation practices between districts, regions, and time period. One of her strategies involved asking a fellow adoptee for their birth certificate so she could try to “match” it while skimming the books on FamilySearch, seeing what book it’s registered in as a shortcut to, hopefully, find more. In addition to seeking historical examples to aid with her sampling strategy, she was also inductively developing a sense of what indicators to look for in order to try to infer whether the subject was adopted – indicators that might necessarily differ depending on the region whose books she was reviewing, or the time period that she was working in. Forrester explained via email in August 2020, drawing on her work experience as an early years educator, “I feel like I am getting to grips with the 1980s layout [of birth registries] and recognizing patterns. The closest example is: when a child who is learning to count recognizes the number names, then counts using 1–1 recognition and then suddenly immediately recognizes he knows the quantity is number 4 without having to count.” Her fine-grained consideration of the documents themselves is thus one element of her scientific labor.

Two examples follow.^15^ Figure 1 shows a blank, voided 1983 birth registration form from the Municipality of Metropolitan Lima. The actual certificates are filled with handwritten text that Forrester (or an anonymous indexer) would try to decipher. Forrester looked at several key indicators to quickly determine whether the registration was an adoptee’s. First, the text running vertically down the page reads “Registry of birth by court order.” This indicates that the entry is somehow exceptional – legal, rather than having been registered due to a hospital or home birth. Another clue to this origin would be found when the pre-printed text “Primera Instancia” (trial court) is crossed out, or redacted, and “Menores” (juvenile court) hand-written above it as the instigator of the birth’s registration. Second, Forrester would look at the parents’ surnames and first names – last names like Rossi or Barbieri might make her think the parents were from Italy, while a last name like Van Dijk might make her think the parents were Dutch. Lastly, she would look for a substantial gap between the birth date (noted on the top left margin under “fecha” and partway down the page where it states that the birth “took place in this city on the following date … ”) and the date that the document was recorded. That latter date is another area where the text is often lightly modified (at the top, “Today, at X time on X day of X day of X month”), indicating a difference between a prescribed format focusing on recording a birth in the month that it happened, and the actuality of recording a child’s new legal origin in a month that may differ from the month of birth.

For comparison, Figure 2 shows a blank, voided 1990 birth registration form, also from Lima. Physical differences in the two documents’ properties can easily be seen – a different layout orientation, a different font, and some difference in the information sought. When reviewing certificates from this decade, Forrester might look to slightly different indicators. The blank spot in the left margin (headed “declarante identificado con:”) would be used to write “court order file [number],” and sometimes the location of the original birth certificate is mentioned, creating an unauthorized potential link between old and new identities. The parents’ nationality is now requested, and anything other than *peruana* would pique Forrester’s interest. While “foreignness” of parents’ surname was no longer a reliable clue (adoptions had diversified by this point to include Spain and South American countries), the blank “domiciliado en” (residing at) might be filled with a hotel name and address. The text “El declarante” might then be bracketed out, followed instead by the date of the court order, the kind of court (juvenile), the judge’s name, and the date that the certificate was recorded. Again, these dates might differ substantially (by months, if not years).

In sum, the three kinds of indicators Forrester came to recognize as important were: a temporal gap (indicating “late” registration – inscribing the document in a time far removed from the birthdate),^16^ the presence of legal terminology (like “court” or “file” or reference to specific laws),^17^ and markers of apparent “foreignness” for the parents like last name, nationality, passport number, or hotel address.

## 5. Analyzing the data: citizen science

This article also considers Forrester’s synthesis of trends and themes. Drawing on her purposive sample, Forrester conducted a comparative analysis of the regions and decades for which she had gathered data and identified some intriguing differences indicative of a particular approach to documentation of demographic events. The above-noted space for “nationality” on the 1990 certificate, and not the 1983 certificate, is one example. When nationality is not even an empty blank on a birth certificate, it means that the assumption is Peru, and alternatives were not really imaginable. By contrast, an adoptee perusing their own certificate would not have that broader awareness of why “nationality” was absent, or present; as Kim has written, adoptees do not typically have the ability “to read [their] documents socially or historiographically” (2019, 453). Through the collection and review of hundreds of certificates, Forrester built an awareness of what is “missing” from particular ways of recording births, adoptions, and other origins.

Prompted by this special issue, we decided to explore making some broader claims drawing on Forrester’s data, and Forrester took up the challenge of creating visuals to illustrate the potential of her work. One initial finding of interest concerns gender breakdown of the 584 children in her sample who were adopted in metropolitan Lima during a four-year period (1990–1993). We were interested to see that boys and girls were essentially equally represented: 294 of the adoptees were male and 290 female. When considered together with the children’s age, as shown in the first table, this suggests that infants were not placed for adoption because of gender prejudice, but rather other factors such as the economic and social challenges of raising a young child in a precarious political moment in Peru.

Table 1 shows a sub-section of five months of the same Lima sample from August to December of 1990, totaling 100 birth registries, broken down by the child’s age at the time of adoption. We were both surprised to see the significant overrepresentation of very young children (six months or younger) in these adoptions. In a time prior to the formalization of administrative adoptions (where, as currently, a guardianship investigation must be conducted over the course of at least a couple of months to ensure the child does not have a potential guardian), this raises questions about the speed with which consent was acquired from the birth mother. Forrester, an early years teacher, also considered the significance of age for the developmental stage of the child and its potential relationship to attachment.

Our remaining two tables concern the destinations of adopted children, with adopting parent’s nationality as a proxy for the destination of the child.^18^ Table 2 shows the Lima sample of 584 birth registries (1990–1993), broken down this time by adopting parents’ nationality.^19^ The US has an outsized appearance on this chart, but is also much more populous than the next three largest destinations (Italy, Canada, and France). Selman offers a demographic method for understanding adoption rates as they relate to birth rates, by determining how many intercountry adoptions occurred per 1,000 live births (2002, 211–213). Using this method, the US’s adoption rate is 4.2, Italy’s 3.9, Canada’s 5.2, and France’s 5.3. Applying a similar process to Forrester’s data, the US’s rate of adoptions *from Lima* during this four-year period (1990–1993) is 0.09, Italy’s is 0.13, Canada’s 0.18, and France’s 0.04.^20^ Selman’s method lets us see that while the USA received the vast majority of Limeño infants in this period in terms of bulk numbers, Limeño adoptees were a larger percentage of the new babies in Canada and Italy.

Italy is even more dramatically represented in Table 3, which shows adoptive birth registrations from a selected district of Cusco during two consecutive two-year-inclusive periods: 1988–89 (59), and 1990–1991 (39). In contrast with the diversity of destinations found in the Lima region, this district processed the majority of adoptions during this period to Italy. This data raises new questions in historical demography, about the structural conditions that led to Italy’s overrepresentation here – might there have been a close connection between an orphanage there and a community in Italy, for example, or could this be a kinship-focused form of what demographers call “chain migration” (see also Leinaweaver 2013:110)? Ultimately, graphs like these illustrate the promise of the data Forrester has collected, suggesting the possibility of making grounded demographic claims about adoptive migration (Leinaweaver 2013): who (gender, age, place of origin, etc.) was departing Peru in the last decades of the twentieth century?

## 6. Closing

For Forrester, an Indigenous woman adopted from Peru and raised in the UK where international adoption has been relatively uncommon,^21^ “technology and research has enabled me to bridge the gaps of my knowledge and [learn my] place in the history of adoption.” Her comparative sampling of different documents across regions and time periods gives her “a profound sense of history and perspective” when considering both her own and other adoptees’ personal documents. Her work is a novel challenge to the conventional wisdom on whether, and where, this data can be located. Her own personal investment in the project grounds the patience with which she collects and curates this data. Forrester’s project offers potential insights to both adoptees, who will be able to see themselves and their stories as one part of a larger picture, and adoption professionals, who will begin to grasp the extent of the out-migration (Leinaweaver 2013; Selman 2002) of Peruvian infants and children to families abroad, in a broader context where missing, and violently disappeared, persons are still being sought throughout the region (Baraybar and Blackwell 2014).

Like any scientist, Forrester also has inductively developed a host of new questions – about the relationship between FamilySearch’s parent, the Mormon Church, and the still-majority-Catholic Peru, how this shapes what is available in FamilySearch, and whether a similar project would be possible for other countries where “trámites” (paperwork, bureaucratic forms) do not proliferate to the same extent as they do in Peru. Some of her other questions illustrate how the project links to her personal biography, the “citizen” in the science – for example, observing the unexpected variety in how these “births” are recorded, she asks which way is best and why a particular region would elect to do it one way or another. And she asks how birth mothers knew about adoption, whether they were literate, and the extent to which they understood what they were signing; questions recognizable to adoption studies scholars (e.g. Briggs and Marre 2009, Leinaweaver 2008).

Through this project, Forrester has recognized the fallibility of the documents she and other adoptees avidly seek. For example, she discovered that the space for “birthplace” does not always get filled with a literal birth site, but rather with a place of legal origin (such as the city where a judge finalized an adoption), casting doubt on an element of personal biography that is important for many adoptees’ narratives (see Leinaweaver 2019). As Forrester explains, “As a researcher, I look at this data and think … actually, it’s in the right book, they are – the parents’ names are clear, everything is quite good, but I wonder if [the adoptees] were actually born there. The process was done right, but the truth might not be there.” This resonates with what was expressed by an adoptee quoted by Kim: “I can’t help but feel and wonder if some really, really important bit of information was, I don’t know lost in translation or something” (2019, 452).

Forrester’s encounters with other adoptees have revealed that documents can point to an unexpected range of (or absence of) information. For example, one adoptee’s birth certificate indicates she was born to her adoptive parents – an irregular (but not unheard of; Kane 1993, 334) bureaucratic move that bypassed any adoption process. Another adoptee has many other forms of paperwork but ultimately no birth certificate, which has prevented him being able to get his Peruvian identity card. Adoptees’ differential relationships to documents have effects far beyond the moment of registration, on their present-day citizenship and belonging in the nation of origin. As Kim explains, “the legal technologies that produce adoptable children … also set the terms for adult adoptee citizenship and personhood in a transnational context” (2019, 457). Similarly, Forrester urges readers to consider what a birth certificate, or its absence, means to an adopted person (compare Blyth et al. 2009).

Personally-implicated citizen science can lead both to a democratization of the research process, and to engaged and meaningful findings that reach communities not often heard from in scholarly research. Forrester’s study is grounded in deep awareness of her fellow adoptees’ challenges in finding information about their origins, and ambivalence about the significance of those findings (compare Polleri 2019). When adoptees seek “continuities in their own lives between past, present, and future” (Carsten 2000, 689), the outcomes are individual and specific; yet collectively, “adoptees recognise in each other their common fate, namely that their lives had a contingent origin” (van Wichelen 2019, 350). By situating each person’s personal and individual story in a broader narrative of themes and trends in Peruvian adoption, Forrester aims to “empower the adoptee to go beyond their own personal adoption circumstances and see how they are part of a process and something far bigger.”

## Figures and Tables

**Table 1.** Age of child when (adoptive re-)birth was registered, Metropolitan Lima, August–December 1990 (100 total records).

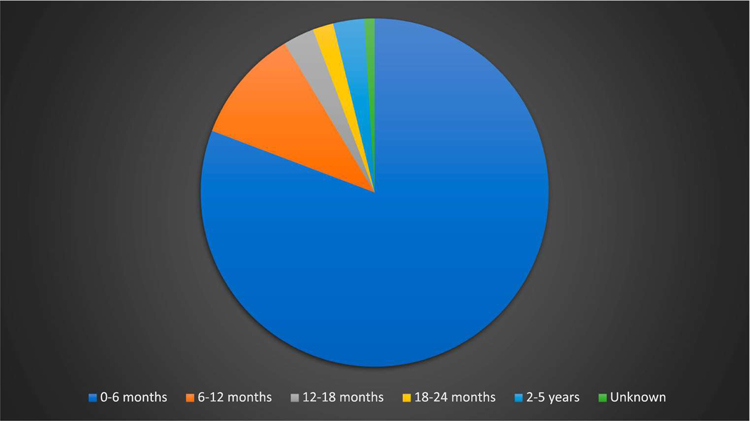

Source: Forrester’s collection.

**Table 2.** Nationality of adopting parents, Metropolitan Lima, January 1990–December 1993 (584 total records).

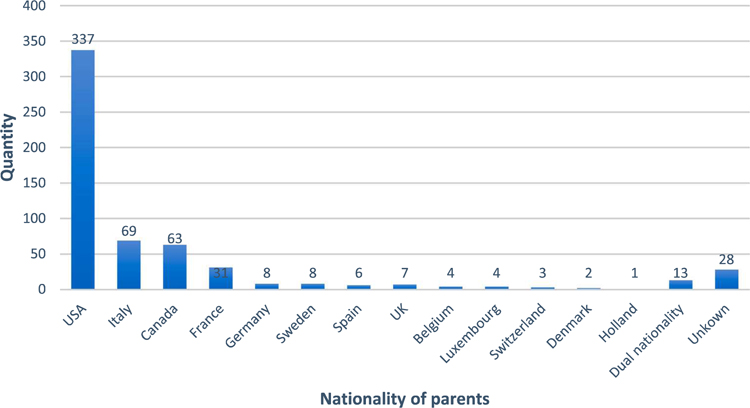

Source: Forrester’s collection.

**Table 3.** Nationality of adopting parents, selected district of Cusco department, 1988–1991 (98 total records).

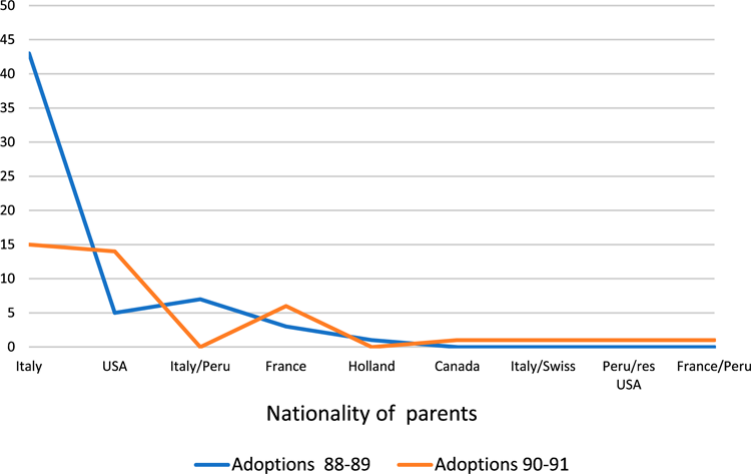

Source: Forrester’s collection.

## Footnotes

1 https://www.familysearch.org/records/archives/web/, accessed 25 September 2021. FamilySearch.org is part of a system that has long been used by historical demographers seeking to analyze changes and continuities over time (see Bean, May, and Skolnick 1978; Otterstrom, Bunker, and Farnsworth 2021, 5; Cherkesly, Dillon, and Gagnon 2019; Oliveira 2018).

2 https://www.gob.pe/529-inscripcion-de-nacimiento-extemporanea, accessed 26 September 2021.

3 RENIEC executive order (Decreto Supremo N° 015-98 PCM, Registration Regulations), Article 22. As an example, in 1994, 21,223 births were registered in metropolitan Lima; 7,382 paternity claims were made; and 179 adoptions were registered (https://www.inei.gob.pe/media/MenuRecursivo/publicaciones_digitales/Est/Lib0012/N64/anexo011.htm, accessed 4 October 2021).

4 No. 010-2005-MIMDES, Article 31; see also Article 39 of the RENIEC executive order, cited above, and Leinaweaver (2008, 51–52). In many contexts adoption requires a series of legal actions to transfer kinship; see Posocco (2011, 444–445) for an example, a 2003 Guatemalan declaration of consent.

5 Forrester contacted Leinaweaver after reading her book (Leinaweaver 2008). We first met in Boston in 2015 and reconnected during lockdown in 2020, meeting by Zoom regularly since then. This article represents the first publication from a novel collaboration between “researcher” and “researched.” Our conversations have been wide-ranging and really interesting for both of us and we look forward to future work together.

6 Some examples of professional demographers trying to make sense of complex data that address reliability and generalizability in this way include Wrigley and Schofield (1981) and Kertzer and White (1994).

7 Forrester had turned to documentation before in her quest to understand her own background; accessing Peru’s RENIEC system (Registro Nacional de Identificación y Estado Civil, i.e. National Registry of Identification and Civil Status; https://www.gob.pe/reniec). RENIEC issued a press release in 2020 describing how it provides access to its archives (https://www.gob.pe/institucion/reniec/noticias/340916-reniec-permite-acceso-a-sus-archivos, accessed 24 September 2021), but clarifies that, per Article 4 of the RENIEC executive order (cited above), certain information is restricted access, including divorce, recognition of paternity, adoption, and declaration of incompetency; see Leinaweaver (2019) on this issue in Spain. Forrester was able to use RENIEC’s archives to access the documents of her birth siblings, and in 2011, the death certificate of her mother.

8 Forrester has gained knowledge and experience with birth searches over many years, and will assist adoptees with searches when possible.

9 For example, including the date of her search would show how long the data collection took, but it also felt like a commemoration. Forrester writes, “As I am recording history and knowing that I will never have this time back nor probably will I have this amount of energy to devote to this act of searching – I felt it was imperative to record the day in which I recorded each search.”

10 Children who are the product of assisted reproductive technologies may also draw on similar DNA technology to learn about their ancestry (Blyth et al. 2009; Herrera, Salvo Agoglia, and Navarro 2019); see van Wichelen (2019, 352) for an analogy for adoptees.

11 Adoption statistics in the UK pose additional challenges. In England and Wales, home studies (prefatory to adoption) are recorded rather than completed adoptions (Selman 2002, 208) and differentiation among international, transracial, and/or relative adoptions is not made (Selman 2000, 17).

12 https://www.familysearch.org/gettingstarted/find-ancestors, accessed 13 July 2021.

13 https://www.familysearch.org/gettingstarted/get-involved, accessed 13 July 2021.

14 Publication in an Open Access journal is a similar kind of project, as Dickel and Franzen (2016, 5) note.

15 A note on method: Although these documents are readily accessible to anyone using FamilySearch, we both feel that reproducing them here would constitute a breach of privacy. Indeed we both share concerns about the range of personally identifiable information available in FamilySearch, which itself is a commentary on the inequality of information access – that FamilySearch has information that is otherwise very difficult to access, which has implications for the use of the data. We considered various methods of emulating the documents – Leinaweaver proposed following Mariner, who, reflecting on Black redaction (e.g. redact this), writes “Adoptees are quite familiar with redaction. It structures our access to certain forms of self-knowledge” (2020, 12). Forrester proposed a different strategy – writing in her own personal information to illustrate (see Mariner 2020, 10). Ultimately we used blank certificates, taken from the end of the scanned books, available instances that were never filled in as the calendar shifted to a new year or the form was replaced with another.

16 Prior to the early 1990s, births registered outside of the period established by law had to be done through “legal order” (mandato judicial), traces of which appear on the documents Forrester reviewed. Law 25025 (1989) made it easier to register births “late.” INEI 1995, “Boletín de Estadísticas Vitales 1994–95, de Lima Metropolitana,” Chapter 2. https://www.inei.gob.pe/media/MenuRecursivo/publicaciones_digitales/Est/Lib0012/C2-1.htm, accessed 26 September 2021.

17 For example, in another series of books, the pre-printed text included the vertical sentence “extraordinary recording per Law 25025” (see note 16). Extraordinary here refers to unusual, out of sequence.

18 A small minority of adopters do not reside in the country of their citizenship.

19 We collapsed all couples with two nationalities (e.g. France/USA) into the category “dual nationality,” and could not estimate the nationality of all parents.

20 This comparison is for illustration only; the match with Selman’s data is not exact, as Forrester’s data are from 1990 to 1993 and Selman’s are from 1997 to 1998.

21 Demographer Peter Selman reports a ratio of 0.5 adoptions per 1,000 live births in 2004 in the UK, compared to 12.40 in one of the “hot spots,” Spain.
